# The World Health Organization Classification of Odontogenic Lesions: A Summary of the Changes of the 2022 (5^th^) Edition

**DOI:** 10.5146/tjpath.2022.01573

**Published:** 2022-05-19

**Authors:** Merva Soluk-Tekkesin, John M. Wright

**Affiliations:** Department of Tumor Pathology, Institute of Oncology, Istanbul University, Istanbul, Turkey; Department of Diagnostic Sciences, School of Dentistry, Texas A&M University, Dallas, Tx, USA

**Keywords:** WHO classification, Odontogenic cysts, Odontogenic tumors, Adenoid ameloblastoma, Surgical ciliated cyst

## Abstract

The 5th edition of the World Health Organization (WHO) Classification of Head and Neck Tumors opened to online access in March 2022. This edition is conceptually similar to the previous classification of odontogenic lesions. The only newly defined entity in odontogenic lesions is *adenoid ameloblastoma*, which is classified under benign epithelial odontogenic tumors. While not odontogenic, the *surgical ciliated cyst* is a new entry to the cyst classification of the jaws. In other respects, a very important change was made in the new blue books that added ‘essential and desirable diagnostic criteria’ for each entity to highlight the features considered indispensable for diagnosis. In this article, we review the *odontogenic tumors* and *cysts of the jaw* sections of the Odontogenic and Maxillofacial Bone Tumors Chapter, outlining changes from the 2017 WHO classification and summarizing the essential diagnostic criteria and new developments.

## INTRODUCTION

The new 5th edition of the “World Health Organization (WHO) Classification of Head and Neck Tumours” is available in a convenient digital format for the first time and opened to online access in March 2022 (1). Actually, this is the first time that the WHO Classification of Tumours Series presents the authoritative content of the tumor classification book series in a digital format. This online version allows the user to access the book anytime and anywhere with electronic devices. Other significant changes made for the first time in the WHO series include the availability of at least one whole slide imaging to significantly improve users’ appreciation of the histologic spectrum of each lesion. Additionally, the addition of essential and desirable diagnostic criteria should improve the user’s ability to interpret and diagnose this area of pathology.

The 2022 5th edition is not conceptually very different from the previous 2017 classification of odontogenic lesions. The odontogenic tumor classification, like earlier editions, is mainly divided into two categories, based on biologic behavior, as malignant and benign. Unlike past editions where malignant odontogenic tumors were discussed first, in the current edition, the odontogenic tumors are still organized by tumor behavior, but the malignant tumors are placed last. Benign tumors are classified into three major categories according to their histogenetic origin; epithelial, mesenchymal and mixed types. The only new entity added to benign epithelial tumors is adenoid ameloblastoma (2). Additionally, subtypes of certain odontogenic tumors and odontogenic cysts are more clearly defined and explained. Some challenging aspects of the 2017 classification still remain uncertain, controversial and debatable, such as the classification of metastasizing ameloblastoma, ameloblastic fibroodontoma/dentinoma, and the mural type of unicystic ameloblastoma (3,4). The odontogenic cyst classification, which was removed in the 2005 3rd edition and added in 2017 4th edition, continues in the new edition with the same entities. Surgical ciliated cyst, not a new entity but new to the classification, has been added to cysts of the jaws.

The aim of this review is to discuss updates in the new 2022 WHO odontogenic lesions classification, outlining changes from the 2017 WHO classification and summarizing the essential diagnostic criteria and current molecular advances. Although not an odontogenic cyst, we will also emphasize the new entry of surgical ciliated cysts to the cyst classification of the jaws and nasopalatine duct cyst. Table 1 summarizes the current classification of odontogenic tumors and cysts of the jaws (1).

**Table 1:** 2022 WHO classification of odontogenic tumors and cysts of the jaws.

**ODONTOGENIC TUMOURS**
***Benign epithelial odontogenic tumours*** Adenomatoid odontogenic tumour Squamous odontogenic tumour Calcifying epithelial odontogenic tumour Ameloblastoma, unicystic Ameloblastoma, extraosseous/peripheral Ameloblastoma, conventional Adenoid ameloblastoma Metastasizing ameloblastoma
***Benign mixed epithelial & mesenchymal odontogenic tumours *** Odontoma Primordial odontogenic tumour Ameloblastic fibroma Dentinogenic ghost cell tumour
***Benign mesenchymal odontogenic tumours*** Odontogenic fibroma Cementoblastoma Cemento-ossifying fibroma Odontogenic myxoma
***Malignant odontogenic tumours*** Sclerosing odontogenic carcinoma Ameloblastic carcinoma Clear cell odontogenic carcinoma Ghost cell odontogenic carcinoma Primary intraosseous carcinoma, NOS Odontogenic carcinosarcoma Odontogenic sarcomas
**CYSTS OF THE JAWS**
Radicular cyst Inflammatory collateral cysts Surgical ciliated cyst Nasopalatine duct cyst Gingival cysts Dentigerous cyst Orthokeratinised odontogenic cyst Lateral periodontal cyst and botryoid odontogenic cyst Calcifying odontogenic cyst Glandular odontogenic cyst Odontogenic keratocyst

## ODONTOGENIC TUMOURS

Table 2 highlights the essential diagnostic criteria along with age, gender, localization preference of all odontogenic tumors.

**Table 2:** Age, gender, localization preferences, and essential diagnostic criteria of odontogenic tumors, modified from the 2022 WHO classification (1).

**Odontogenic Tumors**	**Age/Gender/Localization**	**Essential Diagnostic Criteria**
Adenomatoid odontogenic tumor *(* Figure 1 *)*	- 2nd-3rd decades - Female - Anterior maxilla - Pericoronal	- Site in alveolar processes of jaws - Epithelial nodular structure - Rosettes of spindled to columnar epithelial cells - Duct-like structures - Minimal stroma
Squamous odontogenic tumor	- Mean age at diagnosis is 34.8 - No gender predilection - Anterior maxilla and posterior mandible	- Site in tooth bearing areas of jaw - Closely packed islands of cytologically bland epithelium - Uniform squamous differentiation without significant keratinization - No peripheral palisading and stellate reticulum
Calcifying epithelial odontogenic tumor *(* Figure 2 *)*	- 4th decade - No gender predilection - Body of the mandible	-Tooth-bearing areas of the jaws - Sheets, islands and cords of polyhedral cells with distinct cell borders - Very few or no mitoses - Amyloid present
Ameloblastoma, unicystic	- 2nd decade - Slightly male - Posterior body of mandible and ramus	- Single cyst - Ameloblastoma-like epithelial lining
Ameloblastoma, extraosseous	- 5th-7th decades - Slightly male - Soft tissue of mandibular premolar and maxillary molar regions	- Site in gingiva or edentulous alveolar mucosa - No intraosseous component - Histopathologic features as conventional ameloblastoma
Ameloblastoma, conventional *(* Figure 3 *)*	- 4th-5th decades - No gender predilection - Posterior molar site of mandible	- Islands/strands of odontogenic epithelium bounded by cuboidal/columnar cells with palisaded, hyperchromatic nuclei - Reverse polarity - Loose central epithelium resembling stellate reticulum
Adenoid ameloblastoma *(* Figure 4 *)*	- 4th decade - Slightly male - No site predilection	- Ameloblastoma-like component; duct-like structures - Whorls/morules - Cribriform architecture
Metastasizing ameloblastoma	- A mean age 45 years - Slightly male - Primary tumor site: mandible - Metastatic site: lung	Both in primary tumor and metastatic tumor: - Benign conventional ameloblastoma - No cytological atypia or features of malignancy
Odontoma -Complex (CxO) -Compound (CdO)	- 2nd-3th decades - No gender predilection - Posterior body of the mandible for CxO - Anterior maxilla for CdOs	CxO: - Conglomerate mass of enamel and dentin CdO: - Multiple, small tooth-like structures
Primordial odontogenic tumor	- 1st-2nd decades - Slightly male - Posterior mandible	- Mass of myxoid dental papilla-like tissue - Entire periphery covered by columnar or cuboidal enamel epithelium
Ameloblastic fibroma	- 1st-2nd decades - Slightly male - Posterior mandible	- A well-defined and corticated radiolucency - Bland hypercellular, dental papilla-like mesenchyme - Dispersed bilaminar strands of cuboidal or columnar odontogenic epithelium
Dentinogenic ghost cell tumor	- 3rd- 5th decades - Male - Almost equally in the maxilla and mandible (posterior regions in both jaws)	- Solid tumor - Conventional ameloblastoma-like epithelium - Ghost cells - Dentinoid
Odontogenic fibroma	- A mean age of 34 years - Female - Slightly maxilla (anterior to the first molar)	- Site in tooth bearing segments of the jaws - A well-defined lesion radiologically - Bland fibrous connective tissue of varying cellularity - Varying amounts of odontogenic epithelium
Cementoblastoma	- 2nd-3rd decades - No gender predilection - Posterior mandible (the apical third of permanent first molar)	- Mass fused to a tooth root - Densely mineralized - Radiating peripheral matrix - Plump cementoblasts - No fibro-osseous component
Cemento-ossifying fibroma *(* Figure 5 *)*	- 3rd -4th decades - Female - Premolar and molar region of mandible	- Site in tooth bearing region of jaws - Benign fibro-osseous histology - Well demarcated
Odontogenic myxoma *(* Figure 6 *A-B)*	- 2nd-3rd decades - Female - Premolar or molar region of mandible	- Site in tooth-bearing segments of jaws - Myxoid stroma with variable collagenization - Sparse stellate or spindle shaped cells
Sclerosing odontogenic carcinoma	- 5th-7th decades - Slightly female - Posterior mandible	- A poorly defined radiolucency - Thin cords and nests of epithelium - A dense, fibrocollagenous sclerotic stroma
Ameloblastic carcinoma *(* Figure 7 *)*	- A median age of 49 years - Male - Posterior mandible	- A poorly defined lesion radiologically - Histological resemblance to ameloblastoma with cytological atypia - Features of malignancy
Clear cell odontogenic carcinoma	- A mean age: 53 years - Female - Mandible (posterior body-lower ramus)	- Site in jaws and ill-defined radiolucency - Prominent clear cell phenotype - Infiltrative margin - Exclusion of metastatic disease
Ghost cell odontogenic carcinoma	- 4th-7th decades - Male - Maxilla	- Poorly demarcated lesion radiologically - Ameloblastoma-like epithelium - Prominent ghost cells - Cytological evidence of malignancy
Primary intraosseous carcinoma, NOS *(* Figure 8 *)*	- The mean age: 55-60 years - Male - Mandible (posterior body and ramus)	- Destructive central jaw lesion - Absence of a communication with the surface mucosa or antrum - Exclusion of metastatic disease
Odontogenic carcinosarcoma	- No age incidence - Male - Posterior mandible	- Poorly demarcated lesion in tooth bearing segment - Carcinoma and sarcoma components - Significant cytologic atypia in both components - Exclusion of spindle cell carcinoma.
Odontogenic sarcomas	- The average age: upper 3rd decade - Male - Posterior mandible	- Origin in tooth bearing segment of jaws - Mixed odontogenic neoplasm - Cytologically bland epithelial component - Cytologically malignant ectomesenchymal component

**Figure 1:**
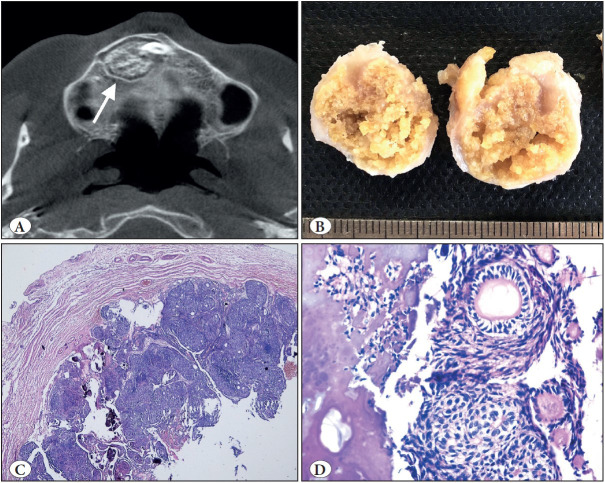
Adenomatoid odontogenic tumor. **A)** Axial CBCT view of right maxillary unerupted canine region showing well-defined lesion with visible internal mineralization (arrow). **B)** Macroscopic appearance of the same case; rounded masses showing a solid yellowish pattern on the cut surface. **C)** Tumor demonstrating a fibrous capsule with odontogenic epithelium in solid nodules (H&E; x40). **D)** At high power, duct-like structures and calcifications clearly seen (H&E; x200).

**Figure 2:**
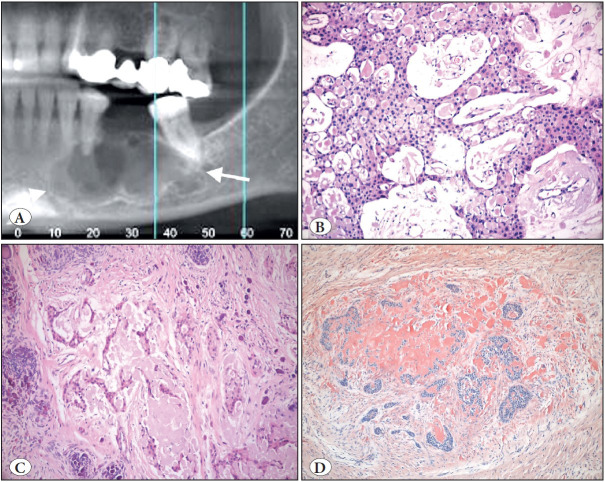
Calcifying epithelial odontogenic tumor. **A)** Cropped panoramic radiograph showing well-defined radiolucency in the left body of the mandible (arrows). **B)** Epithelial sheets composed of polygonal cells with mild nuclear pleomorphism (H&E; x100). **C)** Islands of odontogenic epithelium with focal calcification and amyloid (H&E; x100). **D)** Congo red stain highlights the amyloid material that showed apple green birefringence with polarization microscopy-not illustrated (Congo Red; x100).

**Figure 3:**
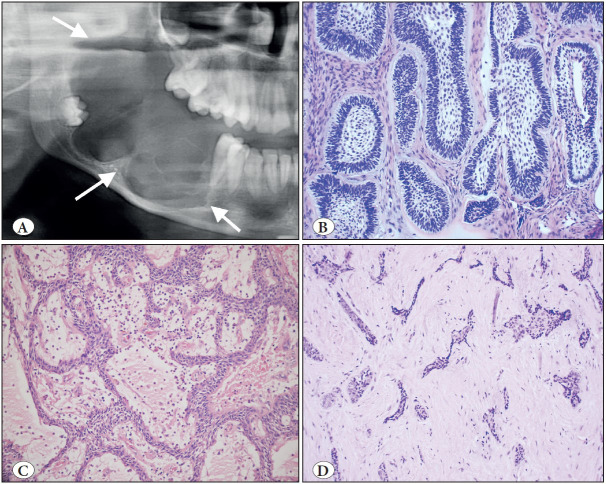
Ameloblastoma. **A)** Cropped panoramic radiograph showing a typical expansive, multilocular radiolucency (arrows). **B)** Follicular pattern; islands where peripheral cells show hyperchromatic nuclei in a palisading pattern, reserve polarity and looser stellate reticulum-like or squamous change in the center (H&E; x200). **C)** Plexiform pattern; anastomosing cords and strands of epithelium (H&E; x100). **D)** Desmoplastic pattern; epithelial islands in dense stroma (H&E; x100).

**Figure 4:**
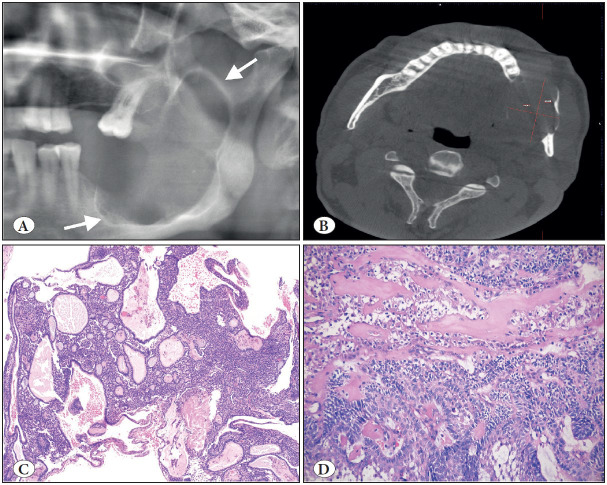
Adenoid ameloblastoma. **A)** Cropped panoramic radiograph showing radiolucent and unilocular lesion with well-defined boundaries (arrows). **B)** Axial CBCT view of the right posterior mandible and ramus showing cortical perforation. **C)** Characteristic cribriform architecture with pseudocysts, duct-like structures and whorls (H&E; x40). **D)** Duct-like clear cells associated with dentinoid matrix (H&E; x200).

**Figure 5:**
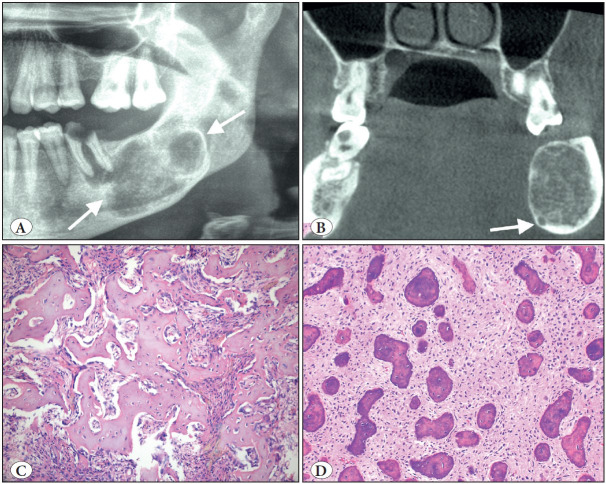
Cemento-ossifying fibroma. **A)** Cropped panoramic radiograph showing a well-defined, expansile radiolucency in the posterior mandible (arrows). **B)** Coronal CBCT view showing the expansion and displacement of the inferior mandibular canal (arrow). **C)** COF is a prototype benign fibro-osseous jaw lesion. The matrix produced can be trabecular with cellular inclusions and osteoblastic rimming like bone (H&E; x200) or **D)** COF often contains smaller rounder and acellular matrix similar to cementum (H&E; x200).

**Figure 6:**
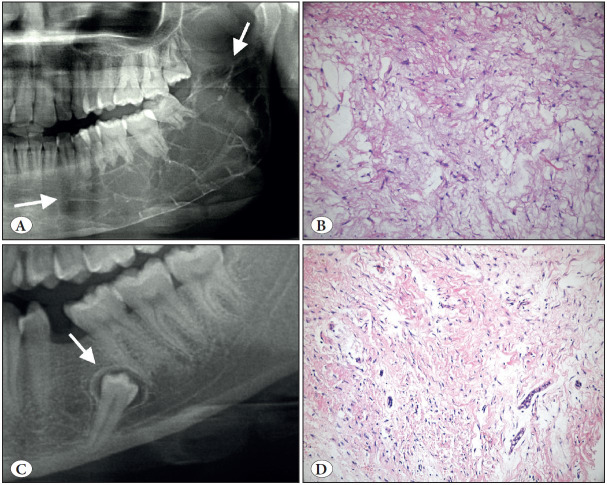
Odontogenic myxoma (A-B) vs. Dental follicle (C-D). **A)** Cropped panoramic radiograph showing the characteristics straight criss-crossing bony septa (arrows). **B)** Odontogenic myxoma; Loose myxoid tissue stroma with scattered spindle and stellate cells (H&E; x200). **C)** Cropped panoramic radiograph showing small radiolucency around the unerupted second premolar tooth (arrow). **D)** Please note the histopathologic similarity with B; there are also some rests of odontogenic epithelium that can also be seen in odontogenic myxoma (H&E; x200).

**Figure 7:**
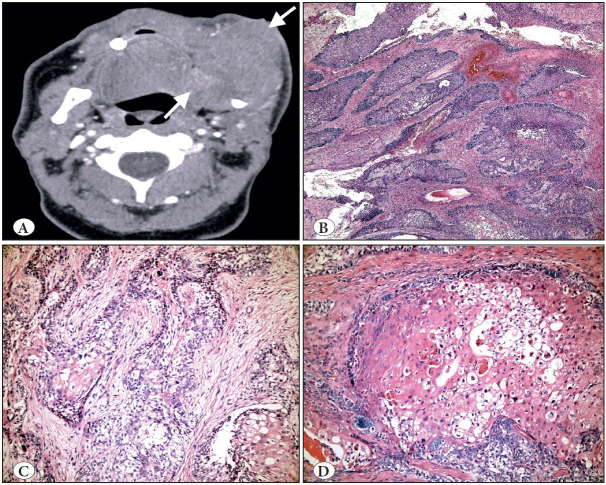
Ameloblastic carcinoma. **A)** Axial CBCT view showing marked expansion, cortical destruction and soft tissue extension (arrows). **B)** Follicular growth where the tumor islands resemble those of ameloblastoma (H&E; x100). **C)** Neoplastic cells displaying significant cytologic atypia (H&E; x200). **D)** Marked atypia, dyskeratosis and clear cell change (H&E; x200).

**Figure 8:**
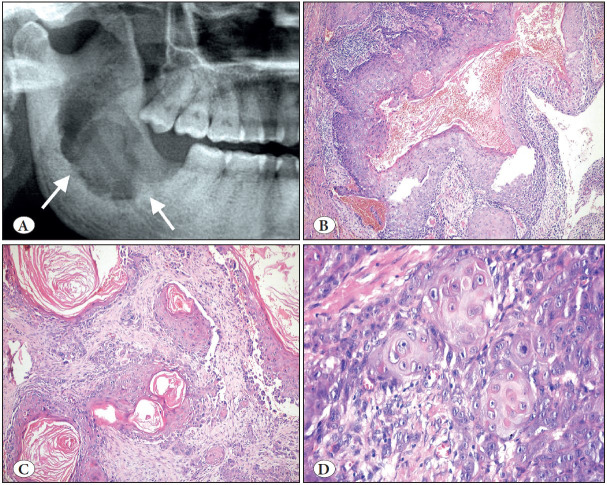
Primary intraosseous carcinoma-NOS arising from a keratinized odontogenic cyst. **A)** Cropped panoramic radiograph showing ill-defined radiolucency in the left mandibular ramus (arrows). **B)** Low power shows the architectural features of a cyst (H&E; x200). **C)** Higher powers show an invasive component with dyskeratosis (H&E; x200). **D)** The invasive component with cytologic features of malignancy (H&E; 400).

### Benign Epithelial Odontogenic Tumors

*Adenomatoid odontogenic tumor* (AOT) has molecular updates and a detailed differential diagnostic section. It is emphasized that some odontogenic lesions, such as odontomas, adenoid ameloblastoma (new entity), adenomatoid odontogenic hamartoma, and adenomatoid dentinoma (the last two not being included in the 2022 classification) may contain AOT-like areas (5,6), and conversely AOT can include calcifying epithelial odontogenic tumor-like areas (7). To avoid misdiagnosis due to the histopathologic overlapping, detailed clinic-radiologic evaluation is necessary as with all bone lesions. Regarding the molecular profile, *KRAS *mutations and MAPK pathway activation are the most common features of AOT that shows *KRAS* p.G12V and p.G12R mutations in about 70% of cases (8).

*Squamous odontogenic tumor* has no major changes from the previous edition.

*Calcifying epithelial odontogenic tumor* (CEOT) has relatively important changes in that three subtypes have been described as clear cell CEOT, cystic/microcystic CEOT, and non-calcifying/Langerhans cell-rich CEOT (1). In the previous edition, these different variants were explained in the histopathological and macroscopic features sections, but they were not included as separate subtypes (4). However, no clear differential diagnostic criteria are proposed to separate the non-calcifying/Langerhans cell-rich CEOT from the amyloid-rich subtype of odontogenic fibroma. These are likely the same tumor but are classified as CEOT by some and as central odontogenic fibroma by others. However, most cases are more suitable for amyloid-rich odontogenic fibroma in terms of morphological and molecular features (9). Although different mutations (*PTCH1, ABMN, PTEN, CDKN2A, JAK3, MET*) have been reported in studies for CEOT, none of them currently affect treatment and diagnosis (1).

*Ameloblastoma, Unicystic*, still has three subtypes according to the distribution of the proliferation of the ameloblastomatous epithelium: luminal, intraluminal and mural. There is general agreement that the first two subtypes can be treated conservatively, but the last one might need to be treated as ameloblastoma (10). The debate continues on whether the mural type is a type of conventional ameloblastoma or not. However, in the new and previous edition it was left under unicystic ameloblastoma.

*BRAF p.V600E* mutations have been found in all types (11). The mural type seems to be somewhere in between conventional and unicystic ameloblastoma in terms of recurrence and might require consideration of more extensive surgery for aggressive and destructive lesions (12). In addition, *BRAF*-targeted therapy can be a consideration for the treatment of mutation-positive cases (13).

*Ameloblastoma, Extraosseous/peripheral* still has a separate entity status while other peripheral odontogenic lesions do not have such distinction, but this has not changed from previous editions. Peripheral ameloblastoma is explained in detail without any major changes from the previous edition.

*Ameloblastoma, Conventional,* the most common odon-togenic neoplasm, excluding odontoma that is considered a hamartoma, is a benign but locally infiltrative neoplasm composed of ameloblast-like cells and stellate reticulum (1). In the 2017 edition, the genetic profile of ameloblastoma was updated broadly. *BRAF p.V600E* is the most common activating mutation, affecting the MAPK pathways, and is an early and critical event in the etiopathogenesis of ameloblastoma (14,15). *BRAF *inhibitor therapy has been proposed for selective cases in the treatment (13,16). It can be predicted that data on these target therapies will increase and will be included in the next classification.

*Adenoid ameloblastoma *(AA) is the only new entity added in the odontogenic lesions and it represents the most important change. It is defined as an epithelial odontogenic neoplasm composed of cribriform architecture and duct-like structures, and frequently includes dentinoid (Figure 5C-D). Approximately 40 cases have been reported in the literature so far (17). It usually presents as a painless swelling with an incidence peak in the 4th decade, and with slight male preference (5,17). The essential diagnostic criteria have been described as an ameloblastoma-like component, duct-like structures, whorls/morules, and cribriform architecture, while dentinoid, clear cells, focal ghost-cell keratinization are reported as desirable features (1). Variable staining for CK14, CK19, p40, p16 and p53 has been reported (18). Ki-67 proliferation index is usually high and that can explain the local aggressive behavior with a high recurrence rate (45.5%-70%) (5,17). Interestingly, in contrast to other intraosseous ameloblastomas, *BRAF p.V600E* mutations have not been found in AA. The lack of BRAF reactivity and the finding of nuclear β catenin reactivity in these tumors calls into question their relation to ameloblastoma and whether adenoid ameloblastoma is the best designation for this new tumor. The main differential diagnosis for AA includes AOT and DHGT but these tumors do not show the essential criteria of AA. Clear cell odontogenic carcinoma and odontogenic carcinoma with dentinoid are also in the differential diagnosis, but the first tumor includes *EWSR1* rearrangement, while distinguishing AA from the latter can be difficult as there are few clear distinctions so far (1).

*Metastasizing ameloblastoma, *defined as an ameloblastoma that has metastasized despite its benign histopathological appearance (1)*, *is still controversial in terms of its classification. This tumor, which was classified under the odontogenic carcinoma section in 2005 (19), was classified under the benign epithelial odontogenic tumors in 2017 (4), as well as in the current edition. The challenge is characterizing metastasizing ameloblastoma at the molecular level and whether its genotype is sufficiently distinct to allow metastasis despite its bland morphology histologically. The solution of the classification issue, which contradicts its name, seems to be left to the next classification.

### Benign Mixed Epithelial & Mesenchymal Odontogenic Tumors

*Odontoma* is now considered a hamartomatous odontogenic lesion with compound and complex types. The section has a very detailed discussion about ameloblastic fibroodontoma (AFO) and ameloblastic fibrodentinoma (AFD), which were excluded from the 2017 and current classification because most examples were presumed to represent developing odontomas. However, the presence of *BRAF p.V600E* mutations in AFD and AFO similar to ameloblastic fibroma, but different from odontoma, has supported the arguments that at least some of these lesions are in fact neoplastic, particularly those with a locally aggressive biological behavior, large size, and recurrence (20,21). A recent study suggests that the combination of age and lesion size may be used to distinguish between lesions of a true neoplastic nature (i.e., AFO) and hamartomatous formation (i.e., OD) (22). On the other hand, it is obvious that we need further molecular and genetic specifications to better understand their true nature.

*Primordial odontogenic tumor*, a new entity to the 2017 classification does not have any significant changes due to its rarity.

*Ameloblastic fibroma* also has a detailed discussion about the relationship of AF with AFO, AFD, and odontoma in the histopathology and pathogenesis sections. Both odontoma and ameloblastic fibroma sections of the new edition have a discussion about AFO and AFD compared to previous editions, and this may indicate a need for further clarification of these lesions in the next classification.

*Dentinogenic ghost cell tumor* is a rare benign odontogenic tumor, which kept its entity status without any important updates.

### Benign Mesenchymal Odontogenic Tumors

*Odontogenic fibroma* now clearly has subtypes; amyloid subtype, granular cell subtype, ossifying subtype, and hybrid odontogenic fibroma with central giant cell granuloma (1). Amyloid type characterized by amyloid deposits with Langerhans cells is a well-known entity but a controversial tumor as pointed out earlier in CEOT and needs to be classified as a CEOT or odontogenic fibroma, not both.

*Cementoblastoma* now has some molecular updates that it shows c-FOS overexpression and harbors the same FOS rearrangement (23) as osteoblastoma, a histologic mimicker. This raises the question of cementoblastoma being a unique odontogenic tumor or a bone neoplasm simply within the spectrum of osteoid osteoma and osteoblastoma.

*Cemento-ossifying fibroma (COF)* was classified under mesenchymal odontogenic tumors in the 2017 classification for the first time but discussed in detail with the other ossifying fibromas in the fibro-osseous lesions section (4). While COF has always been a benign fibro-osseous lesion as well as an odontogenic neoplasm, it is now defined and updated under the odontogenic tumor section. A variety of infrequent molecular alterations have been reported in COF but no pathogenic alterations were identified when 50 oncogenes or tumor suppressor genes were examined by NGS (24).

*Odontogenic myxoma* (OM) has some updates in its pathogenesis that shows MAPK/ERK pathway activation, and this pathway inhibition may have the potential to reduce tumor growth (25). It is worth emphasizing, as the previous edition did, that the most important differential diagnosis of OM is the dental papilla of a developing tooth or a normal/hyperplastic dental follicle that is almost identical histologically to OM (Figure 6C-D), but familiarity with these anatomic structures and the clinical and radiologic features will avoid misdiagnosis (Figure 6).

### Malignant Odontogenic Tumors

There are not many significant differences between the last two editions of the Blue Book in terms of histopathological description and classification of malignant odontogenic tumors. Due to the lack of defined UICC staging guidance, the use of International Collaboration on Cancer Reporting minimum data set reporting is encouraged for all malignant odontogenic tumors (26).

*Sclerosing odontogenic carcinoma* is a very rare odontogenic carcinoma that was added to the classification in 2017 (4). Due to the rarity of cases, there are no molecular updates or developments. As a ‘minor’ change, not having the potential for metastasis was added to the definition of SOC, which was characterized by bland cytology, markedly sclerotic stroma and locally aggressive infiltration (1). Because the original publication (27) suggested and the WHO now concurs that this neoplasm has “no metastatic potential”, should it be categorized as a carcinoma?

*Ameloblastic carcinoma* has a definition change from the previous edition that was accepted and described as a malignant counterpart of ameloblastoma (1). Now, it is considered a primary odontogenic carcinoma histologically resembling ameloblastoma. *BRAF p.V600E* mutations, the most common activating mutation in conventional ameloblastoma, have been reported in AC (28) but it has no defined diagnostic value yet.

*Clear cell odontogenic carcinoma* has no significant changes. It is well known that more than 80% of cases harbor a translocation involving *EWSR1* and *ATF1* (29), a common pathogenesis also of hyalinizing clear cell carcinoma (30). The differential diagnosis is broad and includes almost all clear cell rich tumors, including odontogenic tumors, salivary gland tumors, and metastatic tumors, particularly renal cell carcinoma. Distinction may require IHC/molecular studies in some challenging cases.

*Ghost cell odontogenic carcinoma* has limited update on molecular aspects because of the rarity of the tumor. In the new edition, a single case-documented mutation of *CTNNB1 (β-catenin)* (31) has been added to the previous molecular profile including multiple changes in the SHH signaling pathway, and a novel *APC* mutation (32).

*Primary intraosseous carcinoma-NOS* is usually squamous carcinoma with variable differentiation, but mostly moderately. A recent systemic review found that the majority arise from odontogenic cysts, more commonly residual and radicular cysts and less often dentigerous and odontogenic keratocysts (33). This diagnosis should be made carefully after excluding other malignant odontogenic and intraosseous salivary gland tumors, metastatic lesions, and carcinomas invading bone from other anatomic structures.

*Odontogenic carcinosarcoma* is an extremely rare malignant odontogenic tumor containing both malignant epithelial and mesenchymal components that require very careful examination. Any sarcomatoid change in a malignant epithelial odontogenic tumor should be evaluated carefully to avoid misdiagnosis of a spindle cell odontogenic carcinoma (34).

*Odontogenic sarcomas* are a group of malignant odontogenic tumors; ameloblastic fibrosarcoma, ameloblastic fibrodentinosarcoma, ameloblastic fibro-odontosarcoma. At least 24% of ameloblastic fibrosarcomas arise in benign AF or recurrent AF (35). Dentin/dentinoid with or without enamel/enameloid matrix is produced in approximately 10% of cases and designated as ameloblastic fibro-dentinosarcoma and ameloblastic fibro-odontosarcoma, respectively (1).

Odontogenic carcinoma with dentinoid (36), is reported but not included as a separate entity in the 2022 classification. It is mentioned frequently in the differential diagnosis of other odontogenic tumors and its justification and placement in the classification remains controversial but an area in need of clarification.

## CYSTS OF THE JAWS

Table 3 highlights the essential diagnostic criteria along with age, gender, and localization preference of all odontogenic cysts, surgical ciliated cyst and nasopalatine duct cyst. In the 2017 classification, the cysts of the jaws were divided into two primary parts; odontogenic cysts of inflammatory origin and odontogenic/non-odontogenic developmental cysts (4). Now, in the 2022 classification, the umbrella term of ‘cysts of the jaws’ has been used without any subdivision. However, here we prefer to discuss them under the subheadings of *inflammatory odontogenic cysts, developmental odontogenic cysts*, and *other cysts of the jaws *for greater clarity and to emphasize their origin.

**Table 3:** Age, gender, localization preferences, and essential diagnostic criteria of jaw cysts, modified from the 2022 WHO classification (1).

**Cysts of the Jaws**	**Age/Gender/Localization**	**Essential Diagnostic Criteria**
Radicular cyst - Residual cyst *(* Figure 9 *)*	- 4th-5th decades - Slightly male - Anterior maxilla	- Non-vital tooth for radicular cyst - Edentulous area for residual cyst - Non-keratinized stratified squamous lining epithelium
Inflammatory collateral cysts - Paradental cyst (PC) - Mandibular buccal bifurcation cyst (MBBC)	- 4th decades for PC - 1st-2nd decades for MBBC - Male - Mandibular third molars for PC - Buccal aspect of mandibular first or second molars for MBBC	- Associated with partially or recently erupted vital tooth - Radiolucency distinct from dental follicle - Intact lamina dura - Non-keratinized stratified squamous cyst epithelium
Gingival cysts** ** - Adult type - Infant type	- 5th-6th decades for adults - Neonates for infants - No gender predilection -Gingiva of mandibular premolar/canine region for adult type - Anywhere on the edentulous alveolar ridge for infant type	Adults: - Site in attached gingiva - Thin epithelial lining Infants: - Site in alveolar ridge - Less than 3 months (age)
Dentigerous cyst	- 2nd-3rd decades - Male - Third molars	- Well-defined radiolucency associated with the crown of an unerupted tooth - Cyst attached to the cementoenamel junction - Non keratinized stratified squamous lining epithelium without palisaded basal cells
Orthokeratinized odontogenic cyst *(* Figure 10 *A-B)*	- 3th-4th decades - Male - Mandible (angle-ramus region)	- Tooth bearing areas of jaw - Epithelial lining with orthokeratinization
Lateral periodontal cyst/ Botryoid odontogenic cyst	- 5th-7th decades - Male - Canine/premolar region of mandible	- Site on the lateral aspect or between the roots of vital erupted teeth, mandibular cuspid/premolar - Characteristic whorled epithelial plaques - Multilocularity for botryoid odontogenic cyst
Calcifying odontogenic cyst *(* Figure 11 *)*	- 2nd-3rd decades - No gender predilection - Almost equally in the maxilla (strong predilection for the anterior) and mandible	- Cystic architecture - Numerous ghost cells
Glandular odontogenic cyst *(* Figure 12 *)*	- 5th-7th decades - No gender predilection - Anterior mandible	- Radiolucent cystic lesion of tooth-bearing area of the jaw - Epithelial lining of variable thickness (epithelial thickenings, plaques or papillary projections)
Odontogenic keratocyst *(* Figure 10 *C-D)*	- 3rd-4th decades; a second smaller peak in the elderly - Slightly male - Posterior mandible and ramus	- Site in jaws - Parakeratinized epithelial lining - Palisaded hyperchromatic basal cells
Surgical ciliated cyst *(* Figure 13 *)*	- 5th-6th decades - No gender predilection - Posterior maxilla	- A history of previous surgery - Radiolucent well demarcated cyst - Respiratory epithelial lining
Nasopalatine duct cyst	- 4th-6th decades - Male - Midline of the anterior hard palate	- Epicenter at incisive canal (size greater than 6mm) - Lining of non-keratinized squamous or respiratory epithelium

**Figure 9:**
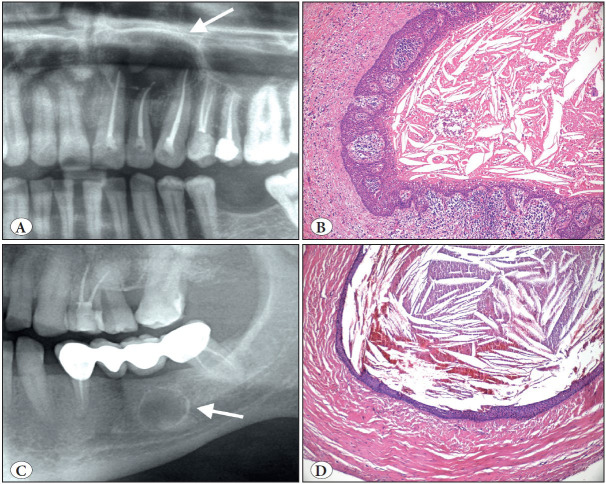
Radicular cyst. **A)** Cropped panoramic radiograph showing a well-defined, corticated unilocular radiolucency at the apices of endodontically treated teeth (arrow). **B)** Lining by non-keratinized stratified squamous epithelium with epithelial hyperplasia in a characteristic arcading pattern. Cyst wall is inflamed (H&E; x100). **C)** Cropped panoramic radiograph of residual cyst showing a well-circumscribed, corticated unilocular radiolucency in an edentulous area of the left mandible (arrow). **D)** Residual (or long-standing) cyst showing less inflamed wall and a more regular thin epithelium (H&E; x100).

**Figure 10:**
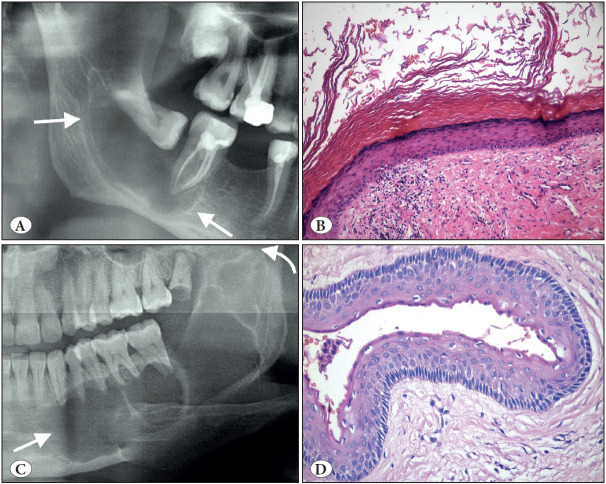
Orthokeratinized odontogenic cyst (A-B) vs. Odontogenic keratocyst (C-D). **A)** Cropped panoramic radiograph showing a well-circumscribed unilocular radiolucency associated with an unerupted third molar (arrows). **B)** OOC is lined by a uniform stratified squamous epithelium with orthokeratosis, prominent granular cell layer and bland, unpalisaded basal cells (H&E; x200). **C)** Cropped panoramic radiograph showing a multilocular radiolucency of the left mandibular body and ramus (arrows). **D)** OKC is lined by a uniform stratified squamous epithelium with a corrugated surface of parakeratin and palisaded and hyperchromatic basal cells (H&E; x200).

**Figure 11:**
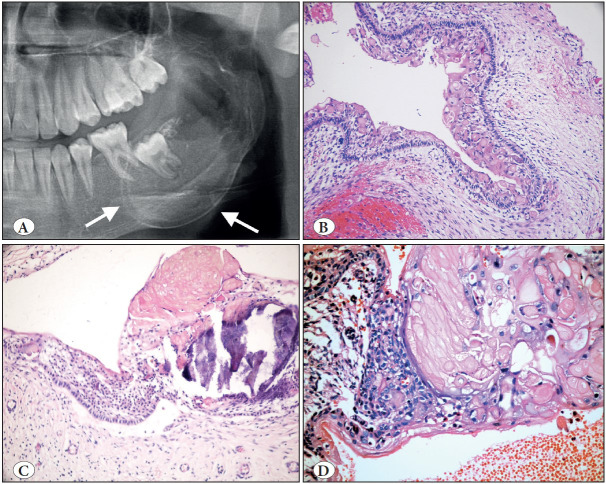
Calcifying odontogenic cyst. **A)** Cropped panoramic radiograph showing well-defined, unilocular, mixed radiolucent/radiopaque lesion with distinct cortical expansion of the left posterior mandible and ramus (arrows). **B)** Low power shows a cystic architecture with prominent eosinophilic, polyhedral cells (ghost cells). (H&E; x200). **C)** Focus of ghost cells, some of which show calcification (H&E; x200). **D)** Characteristic ghost cells where the nucleus is lost but cytoplasmic outlines are maintained (H&E; 400).

**Figure 12:**
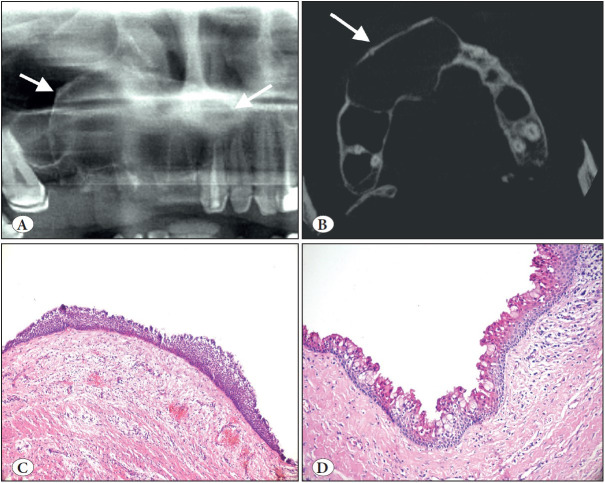
Glandular odontogenic cyst. **A)** Cropped panoramic radiograph showing a large well circumscribed unilocular radiolucency of the right maxilla (arrows). **B)** Axical CBCT view showing significant cortical expansion (arrow). **C)** Cyst lining of variable thickness with enlarged, eosinophilic hobnail cells on the luminal surface (H&E; x100). **D)** Hobnail luminal cells with mucous cells and occasional clear cells (H&E; x200).

**Figure 13:**
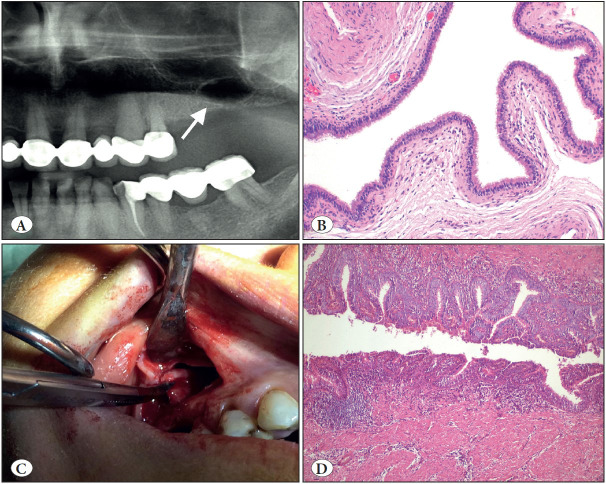
Surgical ciliated cyst. **A)** Cropped panoramic radiograph showing a well-demarcated unilocular radiolucency of the left maxilla with a history of traumatic tooth extraction (arrow). **B)** The cyst lined entirely by respiratory epithelium (H&E; x100). **C)** Intra-operative view of the case located right site of maxilla. **D)** This case shows hyperplastic pseudostratified ciliated columnar epithelium with mucous cells and inflamed cyst wall (H&E; x200). Awareness of this entity prevents misdiagnosis.

### Inflammatory Odontogenic Cysts

*Radicular cyst* (RC) is still the most common cyst of the jaws and accounts for about 60% of all odontogenic cysts (37). RCs arise from periradicular inflammation secondary to the spread of odontogenic infection resulting from tooth devitalization. Residual cyst, which was clearly mentioned as a subtype of RC, is found as a well-defined radiolucency at a site of previous tooth extraction when the RC was not removed when the offending tooth was extracted. Lateral RC terminology of 2017 has been abandoned and only mentioned as RC that can be located on the lateral aspect of the root associated with a lateral root canal (1,4).

*Inflammatory collateral cysts* have no major changes and continue with two distinct subtypes as paradental cyst and mandibular buccal bifurcation cyst. The histology is not specific, and indistinguishable from RC features.

### Developmental Odontogenic Cysts

*Gingival cysts* (adult and infant types), *dentigerous cyst* (eruption cyst, a superficial subtype of dentigerous cyst over an erupting tooth), *orthokeratinized odontogenic cyst, lateral periodontal cyst, *and* botryoid odontogenic cyst* continue in the 2022 classification without important changes from the previous edition. Regarding molecular updates, *BRAF p.V600E* mutations found in ameloblastomas have not been found in dentigerous cysts (38).

*Calcifying odontogenic cyst* (COC) has continued in the cyst classification with an important change in the definition that also affects the diagnostic criteria. In the definition of the 2017 classification, ‘ameloblastoma-like epithelium’ was excluded and COC is now defined as “a developmental odontogenic cyst characterized histologically by ghost cells, which often calcify.” While most COCs still have ameloblastoma-like epithelium, that feature was moved from an essential feature to a desired one (39). Mutations of *CTNNB1 *which encodes β-catenin has been added to the COC pathogenesis (40).

*Glandular odontogenic cyst* (GOC) also has some differences from the fourth edition in terms of diagnostic histopathologic features. In 2017, ten different histopathologic features were described and observation of at least seven criteria was suggested to make a GOC diagnosis (4,41). The new edition emphasized that even if characteristic, not all features are present in all cases but more features provide more confidence in the diagnosis (1). Among these features, the essential criterion is presence of a lining epithelium with varying thickness, but it was stated that hobnail cells are observed in almost all cases and seem to be the most characteristic feature. It was also emphasized in this classification that the most important differential diagnosis of GOC is intraosseous mucoepidermoid carcinoma. Demonstrating the *MAML2* rearrangement for intraosseous MEC is important in the differential diagnosis of these two lesions (42). That is still generally accepted; however, one GOC case has been reported with *MAML2* rearrangement (43). It is believed that this situation needs further studies.

*Odontogenic keratocyst* (OKC) is the most frequently researched cyst due to high recurrence rate, aggressive clinical behavior, and association with the nevoid basal cell carcinoma syndrome. In the 2022 edition, OKC continues in the cyst classification and has the longest section among the cysts of the jaw. There is extensive literature characterizing the molecular landscape of OKC. Most show mutations of the tumor suppressor gene *PTCH 1*, but rarely *PTCH2* or *SUNU* (44–46).

These mutations have fueled the continued spirited debate about whether OKC is a cyst or a cystic neoplasm. In 2005, OKC was changed to a cystic neoplasm and designated a keratocystic odontogenic tumor (19). It was moved back into the cyst category in 2017 (4) and continues as a cyst in the 5th current classification. The debate continues.

### Other Cysts of the Jaws

*Surgical ciliated cyst,* not a new entity but new to the classification, is a rare non-odontogenic cyst lined by respiratory epithelium as a result of the traumatic implantation of sinus or nasal mucosa. Surgical ciliated cyst of the maxilla, postoperative maxillary cyst or (respiratory) implantation cyst are other terms used for this entity. The most common age range is the 5th to 6th decades with no gender predilection (47). As the definition indicates, it occurs most commonly in the posterior maxilla; but very rarely in the mandible due to implantation of sinus epithelium by contaminated instruments or using nasal bone or cartilage with epithelium for augmentation genioplasty (47,48). Histopathologically, the cyst is lined by ciliated pseudostratified columnar epithelium and mucous cells are common (Figure 13B-D). A history of previous surgery is an essential criterion for diagnosis. Treatment is simple enucleation and recurrence is rare.

*Nasopalatine duct cyst* is the most common non-odontogenic cyst of the jaws (about 80%) (37,49). The cyst is lined by non-keratinized squamous epithelium or respiratory epithelium in variable proportion with focal areas of cuboidal, columnar, or ciliated changes. Generally, the neurovascular bundle is seen in the wall of the cyst, but this is a feature of sectioning and this feature is included in the desirable diagnostic criteria (1).

In conclusion, the new 2022 WHO classification of odontogenic tumors and jaw cysts includes some modifications and developments that we briefly summarized. It is hoped that this summary becomes a resource for readers to find recent changes and critical diagnostic information. Lastly, rapid developments in technology and molecular fields signal that the time between the editions of WHO classification of Head and Neck Tumours (1st 1971, 2nd 1992, 3rd 2005, 4th 2017 and 5th 2022) will become shorter, and new classifications seem inevitable in the next 5 years.

## Conflict of Interest

The authors have declared no conflict of interest.
